# Shifts and Trade‐Offs of Ecological Strategy Associated With Species Diversity and Functional Traits During Vegetation Succession Progress in Karst Ecosystem

**DOI:** 10.1002/ece3.72808

**Published:** 2025-12-23

**Authors:** Menglin Lei, Kaiping Shen, Tingting Xia, Xu Han, Hongchun Chen, Yan Zhao, Xihong Yang, Jiahao Xiao, Fulin Wang, Ying Zhao, Weijie Li, Yuejun He

**Affiliations:** ^1^ Forestry College, Research Center of Forest Ecology Guizhou University Guiyang China; ^2^ Key Laboratory of Karst Georesources and Environment, Ministry of Education Guizhou University, College of Resources and Environmental Engineering Guiyang China; ^3^ Guizhou Key Laboratory of Agricultural Biosecurity Guizhou Botanical Garden Guiyang China

**Keywords:** community succession, CSR strategy, degraded vegetation, diversity, functional traits, trade‐off

## Abstract

The CSR framework reflects the ecological strategies of competition (C), stress tolerance (S), and ruderal (R) that plants employ to adapt to disturbances. This framework provides a robust foundation for exploring community assembly and successional dynamics within degraded ecosystems such as karst. However, the influence of functional traits and species diversity on ecological strategic trade‐offs during vegetation succession remains unclear. The space‐for‐time substitution method was employed to examine the influence of functional traits and species diversity on ecological strategic trade‐offs during vegetation succession. Five successional stages were identified in the karst community: herb, herb‐shrub transition, shrubbery, shrub‐tree transition, and Tree forest. For each stage, five 100 m × 100 m plots were established, with each plot containing four 10 m × 10 m subplots, resulting in a total of 25 plots and 100 subplots. Plant leaf traits from 2503 individuals of 160 woody species were measured and quantified for CSR components at individual and community levels. The redundancy analysis (RDA), Mantel tests, and a random forest model were applied to explore associations in CSR strategies, diversity, and functional traits. Our results revealed a strategic trade‐off, with a shift from the dominance of the S strategy to increased contributions from C and R strategies at both individual and community levels as vegetation succession progressed. This transition was closely associated with woody species diversity and leaf macronutrient traits of carbon, nitrogen, and phosphorus. RDA and Mantel test results further indicated that Hill numbers (*q* = 0, *q* = 1), leaf water content, and leaf carbon content were key determinants of ecological strategy shifts. Additionally, the random forest model identified soil pH as the strongest predictor of the C strategy, whereas Hill number (*q* = 0) was the primary determinant of S and R strategies. In conclusion, the dominance of the S strategy in the early successional stages gradually shifted toward C and R strategies during succession progress. There were strategic trade‐offs of CSR associated with species diversity and leaf functional traits, embodied in S relative to C and R strategies.

## Graphical abstract

Strategy shift from strategy S toward C and R; Strategic trade‐offs for degraded vegetation recovery; Strategies associated with diversity and functional traits.

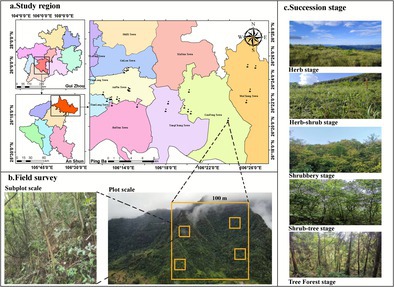

## 1 Introduction

Plants have developed a range of ecological strategies to adapt to selective pressures through long‐term evolutionary processes in heterogeneous environments, ensuring the persistence of populations (Adler et al. 2014). J. P. Grime (1974) proposed the CSR framework, which classifies ecological strategies based on competition (C), stress tolerance (S), and ruderal (R) traits. Specifically, C‐selected species thrive in low‐stress, high‐productivity environments by rapidly acquiring and monopolizing resources. S‐selected species prioritize defense mechanisms to survive in high‐stress, low‐productivity environments, while R‐selected species focus on rapid reproduction through propagule investment in highly disturbed environments (Grime and Pierce 2012). The CSR framework has been widely applied in theoretical and empirical research on species adaptability, vegetation succession, and ecosystem dynamics (Luiza et al. 2015; Rosado and Mattos 2017; Han et al. 2021). Plant functional traits mediate responses to environmental changes, and thus ecological strategies can be effectively inferred from trait‐based approaches (Reich 2014; Chai et al. 2016; Díaz et al. 2016). Furthermore, species diversity is critical in shaping ecological strategies, influencing resource partitioning, niche differentiation, and community stability (Tilman et al. 2014). Therefore, understanding how species diversity and functional traits impact plant ecological strategies is essential for explaining vegetation responses to environmental variation.

Violle et al. (2007) suggested that plant functional traits, including morphological and physiological characteristics, serve as key indicators of evolutionary adaptation. Functional traits can reflect strategic trade‐offs for plants suffering variable environmental pressures (Díaz et al. 2016). Wright et al. (2004) revealed that the leaf economics spectrum (LES), which is a functional combination of traits through a balanced or synergistic variety of leaf traits that could be quantified for plant resource trade‐off strategies. Wang et al. (2024) demonstrated that in evergreen broad‐leaved forests, C‐strategy species adopt an acquisitive strategy with high specific leaf area, nitrogen, and phosphorus, yet low leaf dry matter content. In contrast, S‐strategy species employ a conservative strategy with the opposite trait spectrum. Han, Xu, et al. (2022) found that with increasing woody plant species diversity, the proportion of C‐strategy species increased, while that of S‐strategy species decreased. Han, Huang, and Zang (2022) also found that soil factors (available phosphorus and available nitrogen) are key drivers of the differentiation in CSR strategies among woody plants. However, their research did not explore the differentiation of CSR strategies during the vegetation succession and the driving factors.

Succession is a continuous process of change in plant communities and their environment, with species composition altering over time (Van Breugel et al. 2024). Previous research found that species rapidly colonized habitats to exploit resources in the early stages, favoring high growth rates and resource‐acquisitive traits, while later successional species prioritize lifespan and conservation strategies (Prieto et al. 2016). The increased shrub abundance in the Upper Andean Tropical Forests resulted in a higher community‐weighted mean wood density in early versus late successional forests, and was partially responsible for the decrease in aboveground net primary productivity (Castillo‐Figueroa et al. 2023). These findings indicated that the process of succession led to strategy differences in plant functional traits within the niche space, which were associated with diversity turnover (Poorter et al. 2021, 2023; Castillo‐Figueroa and Posada 2025a). Castillo‐Figueroa et al. (2025) found that functional traits and species identity drive litter decomposition in Upper Andean Tropical Forests, rather than succession and microclimatic conditions of soil moisture and temperature. Pickett (1989) proposed “space‐for‐time substitution” as an alternative to long‐term studies. The space‐for‐time substitution approach is widely employed in contemporary ecology. It underpins important studies across diverse fields, including biodiversity (Addison et al. 2003), nutrient cycling (Vitousek et al. 1995), productivity and carbon flux (Law et al. 2003; Litvak et al. 2003), natural and anthropogenic disturbances (DeLuca et al. 2002), restoration (Aide et al. 2000), and global change (Choi and Wang 2004; Grünzweig et al. 2004). The CSR strategies approach is highly appropriate to understand succession, as it explicitly focuses on plant responses to disturbance (Poorter et al. 2023). Most researchers adopt the space‐for‐time substitution approach to examine CSR strategies during succession, using plant communities from different developmental stages across locations as proxies for temporal sequences (Chai et al. 2016; Mastrogianni et al. 2023). However, there is limited research on the variation of CSR strategies in Karst vegetation succession.

The karst ecosystem, which is typically characterized by degraded vegetation, is widely distributed in southwest China. It generally features sparse plant coverage due to soil erosion, water loss, and exposed bedrock (Zhang et al. 2017). Wang et al. (2023) discovered the strategy differentiation of evergreen and deciduous woody species in association with leaf functional traits in karst forests and suggested the trade‐off between resource acquisition strategy for evergreen plants and resource conservation strategy for deciduous plants. Additionally, Hu et al. (2023) revealed that S‐selection was the dominant strategy relative to C‐selection and R‐selection in karst forests, as stress‐tolerant strategies are better adapted to extreme environments than competitors or ruderals (Rios et al. 2022). However, their study did not explore how biodiversity and leaf functional traits change during the karst vegetation succession.

Zhou et al. (2021) observed a shift from S to R strategy in the communities after rangeland degradation, highlighting that the contrasting strategies of plant species in response to the decrease in available resources might lead to niche expansion of secondary forbs and loss of diversity in the degraded alpine meadows. Vegetation succession typically progresses from herbaceous plants via shrubs to trees, treated as the climax community stage, including some transitional types (Halpern 1988), especially in karst areas. Thus, understanding how woody plants adapt to functional strategy shifts during the progressive succession of degraded ecosystems remains a valuable theoretical endeavor. Indeed, the S‐strategy followed C‐ and R‐dominating karst forests (Hu et al. 2023) can be attributed to a stress‐tolerant strategy that was a better adaptation to extreme environments than competitors or ruderals (Rios et al. 2022). J. P. Grime (1987) proposed a general model of secondary succession in which the ruderals were the start of a community, competitors were dominant at intermediate stages, and stress‐tolerant plants dominated the final community. Under primary succession, the stress‐tolerant strategy was dominant in the early stages, and as succession progressed, the emergence rate of the competitive strategy (C) increased (J. P. Grime 1987, 1988). Existing research indicates that during succession in tropical lowland rainforest, the ecological strategy of woody plant communities shifts from S to C strategy (Chen et al. 2023), and most woody individuals and communities in the karst forest tend to adopt S and C strategies (Hu et al. 2023). Yet, it remains unclear how CSR strategies of woody plants shift during karst vegetation succession, and how these shifts are mediated by leaf functional traits, species diversity, and soil properties. Therefore, we hypothesize that in the early stages of karst vegetation succession, woody plants primarily adopt the S strategy, and as succession progresses, the S strategy of woody plants gradually shifts toward the C and R strategies (H1). Given the central role of plant functional traits in shaping ecological strategic trade‐offs according to Wright et al. (2004) and Pierce et al. (2017), we quantified CSR strategies using key leaf traits of leaf area (LA), leaf dry matter content (LDMC), and specific leaf area (SLA). In addition, according to this research from Wang et al. (2024) and Han, Xu, et al. (2022) ecological strategies related to plant diversity and functional traits, and the reveal of trade‐offs on CSR strategies documented by Hu et al. (2023), we hypothesize that there is a trade‐off between ecological strategies in karst plant communities, which is accompanied by changes in species diversity and plant traits (H2). This study aims to explore CSR ecological strategies concerning plant functional traits and species diversity, providing insights into the adaptive regulation of vegetation restoration in degraded karst ecosystems.

## 2 Materials and Methods

### 2.1 Study Area

**FIGURE 1:**
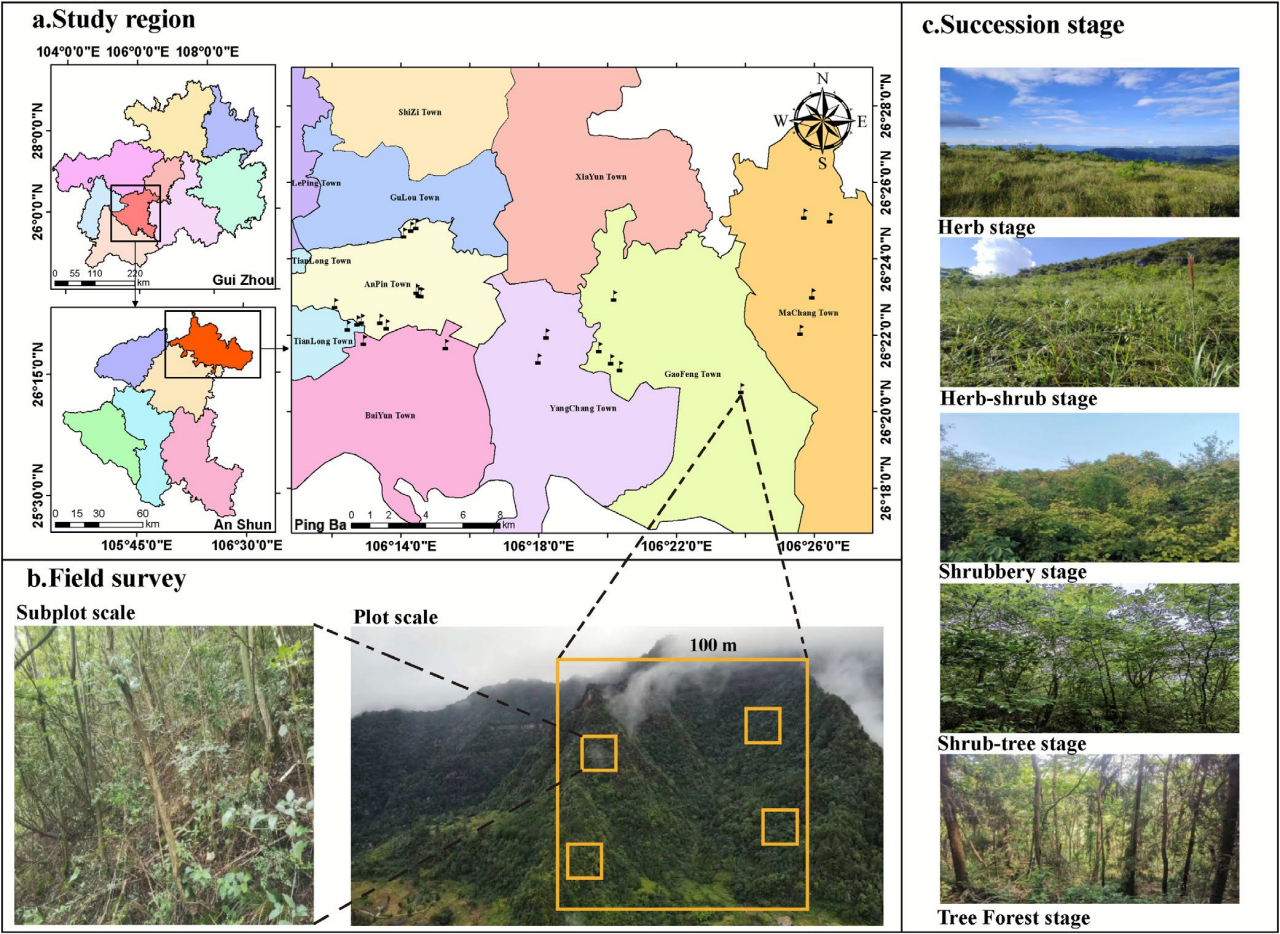
The location sites of surveyed plots and the community pictures from different successional stages. There were 25 plots of 100 m × 100 m divided into 100 subplots of 10 m × 10 m; five sample plots were set up for each stage, with four subplots set up for each plot. These pictures were taken from field surveys, which represent typical plant communities at different stages, including the Herb, Herb‐shrub transition, Shrubbery, Shrub‐tree transition, and Tree forest, using the space‐for‐time substitution approach and the determination from relative abundance and coverage of woody species according to the natural succession principle.

A community survey was conducted in a typical karst landform of Pingba District containing six towns, Guizhou Province, China (106°19′17″–106°26′17″ E; 26°23′28″–26°26′20″ N; altitude: 1239–1324 m; Figure 1), where the subtropical monsoon climate, with annual mean precipitation of 1200 mm and temperatures ranging from 14°C to 24°C. In this study, we employed the approach of space‐for‐time substitution to conduct the plant community survey for different successional stages according to Pickett (1989). In particular, the vegetation was divided into five stages based on vegetation situation and community properties divided by Herb, Herb‐shrub, Shrubbery, Shrub‐tree, and Tree forest, as shown in Table S1, while we assumed that the trajectory and disturbance history were the same at each site in temporal sequence succession, despite some limitations to this method according to Damgaard (2019). In addition, we ensured all sites had environmental consistency in the same climate, soil, and terrain factors. According to our field observations, each stage has distinct characteristics in the species composition, vertical structure in layers and height, life type, and the relative coverage of herbs and woody plants, etc. Of course, we also gathered information on the community's origin from local villagers and the Government Forestry Department to ensure reliable data. Simultaneously, based on information gathered from local villagers and consultations from forestry departments, these plots have not been disturbed by grazing, logging, or other human activities. Furthermore, woody plants occurred in all successional stages from the field observations. In judgment, when the relative abundance of woody plants was approximately < 5% with coverage of < 1%, indicating herb plants dominated the plant community, thus this plot was listed as the Herb stage. Besides, there was a transitive feature composed of herb and shrub plants, approximately shared relative coverage from 40% to 60%, which was listed as the Herb‐shrub stage. If the shrub plants dominate the community and simultaneously differentiate into two layers of community structure, when the average height of the community approaches 1.9 m, which reflects a significant height difference compared to the Herb and Herb‐shrub stages and the Shrub‐tree and Tree forest stages, the community was judged as the Shrubbery stage. Meanwhile, the relative density of tree species was approximately < 10%, and it was also listed as the Shrubbery stage. In the event that the relative density of trees ranges approximately from 10% to 60%, it was categorized into the Shrub‐tree transition; when it exceeds 60%, this community is classified as the Tree‐forest stage. Additionally, the Shrubbery had a typical two‐layer vertical structure; however, an indistinct tree layer with obvious shrub and herb layers occurred in field sites, while the Shrub‐tree stage had obvious three layers in the community structure in field observations. Based on actual conditions, other judgments were referred to Table S1.

### 2.2 Community Survey and Sample Collection

In the field survey, we established a plot of 100 m × 100 m for each stage as five replicates, and we set a subplot of 10 m × 10 m as four replicates in each plot. Consequently, 25 plots and 100 subplots were contained in all stages, see Figure 1. To avoid spatial autocorrelation, we ensured that the distance between duplicate plots was > 200 m in a specific stage and was > 50 m in an established subplot. Subsequently, the plant community was surveyed by established subplots, including the species, individual numbers, plant height, DBH (diameter at breast height for tree species), and the aboveground diameter (< 1.3 m height with ≥ 1 cm diameter). In addition, we collected soil for physicochemical properties, using the five‐point samples method diagonally in all plots (Jiao et al. 2024), with 100 samples from the soil layer with a depth of 0–20 cm in all stages. Furthermore, according to the methods by Pérez‐Harguindeguy et al. (2013), 10 individuals per species were collected from each plot. Where fewer than 10 plants were available, all existing specimens were collected. For each plant, 1–2 branches bearing 5–10 intact positive mature leaves were gathered. Due to practical constraints such as slope gradient and tree height within the plots, consistent sampling of canopy leaves was not always feasible. Therefore, we adopted a flexible sampling protocol: canopy leaves were collected from trees where they were accessible; otherwise, sampling was conducted in the sub‐canopy layer. A total of 2503 woody individuals of 160 species in all plots. All samples of plants and soil were taken into fresh bags and brought back to the laboratory to measure leaf functional traits and soil physicochemical properties.

### 2.3 Measurements for Leaf Functional Traits and Soil Properties

All samples from surveyed plots, including plant and soil, were used to measure leaf functional traits and soil physicochemical properties. On the one hand, leaf traits were measured by the leaf area (LA; cm^2^) using a scanning system by software of WinRHIZO Pro LA2400, the leaf fresh weight (LFW; mg) using a balance of 1/10,000, and the leaf dried weight (LDW; mg) using oven‐dried at 105°C for 48 h to a constant weight. Additionally, the specific leaf area (SLA; cm^2^/g) was calculated by the LA divided by LDW, and the leaf dry matter content (LDMC; %) was calculated by the LDW divided by LFW; simultaneously, the leaf water content (LWC; %) was calculated using the difference of LFW and LDW divided by LFW. Furthermore, the leaf carbon (LC; mg/g) was determined using the potassium dichromate‐sulfuric acid oxidation, and the Kjeldahl method (BUCHI K‐360, Switzerland) and molybdenum antimony methods were used for the measurement of leaf nitrogen (LN; mg/g) and leaf phosphorus (LP; mg/g). At the same time, the stoichiometric ratios of C/N, C/P, and N/P were calculated by their determined concentrations. On the other hand, soil properties were determined by pH indicators, the soil organic carbon (SOC; mg/g), and the total nutrients and available nutrients of nitrogen (N), phosphorus (P), and potassium (K) with the stoichiometric ratios of C/N, C/P, and N/P. These methods of carbon (C), nitrogen (N), phosphorus (P), and potassium (K) indicators of plant and soil mainly referred to methods from Bao (2000) in this study.

### 2.4 CSR Strategy Components and Woody Species Diversity

The CSR strategies were quantified by applying strategy components through the “StrateFy” calculator using traits of LA, LDMC, and SLA referred to by Pierce et al. (2017). The qualified method was adopted widely to explore ecological strategies in various environments (Rosenfield et al. 2019; Liao et al. 2021), due to the reliability of locating CSR strategies for plants (Shipley and Li 2017; Novakovskiy et al. 2021). Initially, the component values of woody plant individuals were calculated using traits of LA, LDMC, and SLA applied in the “StrateFy” spreadsheet for 160 species, representing the individual‐level ecological strategies. Additionally, according to Muscarella and Uriarte (2016), the community‐weighted means (CWM) on leaf traits could reflect community functional traits. Therefore, the CWM traits of LA, LDMC, and SLA at the community level were calculated in association with the relative abundance according to Garnier et al. (2004), using the formula as shown below:

$$\mathrm{CWM}=\sum_{i=1}^{n}\mathrm{RA}\times {\text{trait}}_{i}$$

where CWM is the community‐weighted means, *n* is the number of species in a subplot, and the RA is the relative abundance of species *i*. Subsequently, the integrated CWM traits regarding woody species at the community level were used to obtain community CSR components for every stage through the calculator of “Strategy.” Furthermore, the species diversity of woody plants was calculated by adopting indicators of the Hill numbers (*q* = 0, *q* = 1, *q* = 2) for each subplot (Jost 2006).

## 3 Data Analysis

In data analysis, the CSR component values of individual levels were created ternary plots by integrating the subplot and plot data belonging to different successional stage types by Origin 2021 (OriginLab, Northampton, MA, USA). Strategy component differences of different stages at the community level were analyzed using one‐way ANOVA via SPSS software (version 24.0; NewYork, NY, USA). The regression was performed on community‐level CSR strategy components associated with species diversity of the Hill numbers (*q* = 0, *q* = 1, *q* = 2) and leaf stoichiometric CNP traits by all subplot data using Origin 2021 (OriginLab, Northampton, MA, USA). Further, the strategy components associated with soil properties, leaf functional traits, and species diversity of the Hill numbers (*q* = 0, *q* = 1, *q* = 2) were explored through redundancy analysis (RDA) by using the package “vegan” and Mantel's test by the “linkET” package in R software (v.4.3.1, R Core Team, Vienna, Austria). In addition, the relative cumulative importance of soil properties, leaf functional traits, and species diversity of the Hill numbers (*q* = 0, *q* = 1, *q* = 2) associated with CSR components of the community were analyzed using the random forest model by the “randomForest” package in R.

## 4 Results

### 4.1 CSR Strategies of Plant Individuals and Communities in Different Vegetational Stages

The coefficients of variation (CV) for the community‐weighted means of LA, SLA, and LDMC across all successional stages ranged from 5.27% to 116.01% (Table S2).

**FIGURE 2:**
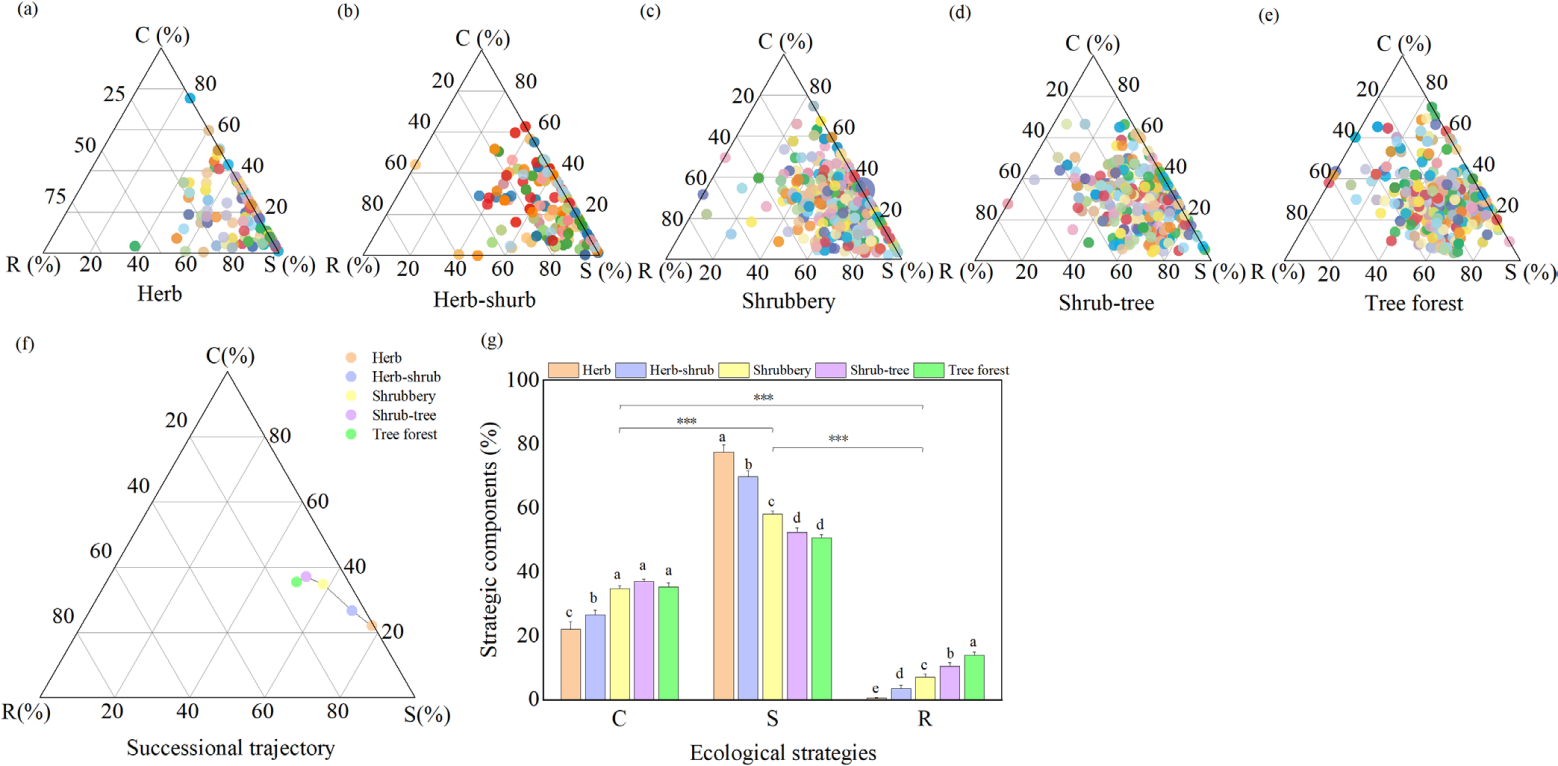
Ternary diagrams of CSR at the individual (a–e) and community levels (f, g) in different succession stages. These circles in ternary diagrams represent different woody plant individuals. Different letters indicate a significant difference in both stages among five successional stages at *p* < 0.05; the *** indicates a significant difference between both components of CSR in total at the *p* < 0.001 level.

Specifically, LA exhibited the highest CV in each stage: 116.01% in Herb, 54.08% in Herb‐shrub, 35.62% in Shrubbery, 35.26% in Shrub‐tree, and 40.63% in Tree‐forest. In contrast, LDMC consistently showed the lowest CV in each stage: 5.27% in Herb, 5.90% in Herb‐shrub, 8.22% in Shrubbery, 12.13% in Shrub‐tree, and 5.66% in Tree‐forest. CSR strategies for 2503 woody individuals from 160 species across five successional stages (Herb, Herb‐shrub, Shrubbery, Shrub‐tree, and Tree‐forest) were quantified based on LA, SLA, and LDMC using the “StrateFy” calculator. The resulting ternary plots (Figure 2a–e) revealed that the circles representing these strategies were primarily clustered on the C–S side, with a clear bias toward the S vertex. At the individual level across all stages, the component S was an average of 59.41% (range: 51.09%–74.22%), followed by component C (average of 26.84%; range: 19.26%–29.88%) and component R (average of 13.75%; range: 6.51%–19.02%; Table S3). At the community level, the successional trajectory of CSR strategies exhibited distinct changes across each stage. Starting from the herbaceous stage, the circles representing the community's CSR strategies gradually shifted away from the S–C side and moved toward the center of the ternary plot as succession progressed (Figure 2f). Meanwhile, the component S was significantly higher than both the components C and R (*p* < 0.001), while the component C was also significantly greater than R (*p* < 0.001); component S gradually decreased as components C and R increased during succession (Figure 2g). These findings demonstrated that the S strategy was dominant during karst vegetation succession, followed by the C and R strategies. This was evidenced by the movement of the majority of circles from the S vertex toward the C–R side in ternary plots across successional stages, indicating a strategy shift from S to C and R. Overall, these results indicated a trade‐off existed between the stress tolerance strategy and the strategies for competition and ruderal during karst vegetation succession.

### 4.2 CSR Strategies Associated With Woody Species Diversity

**FIGURE 3:**
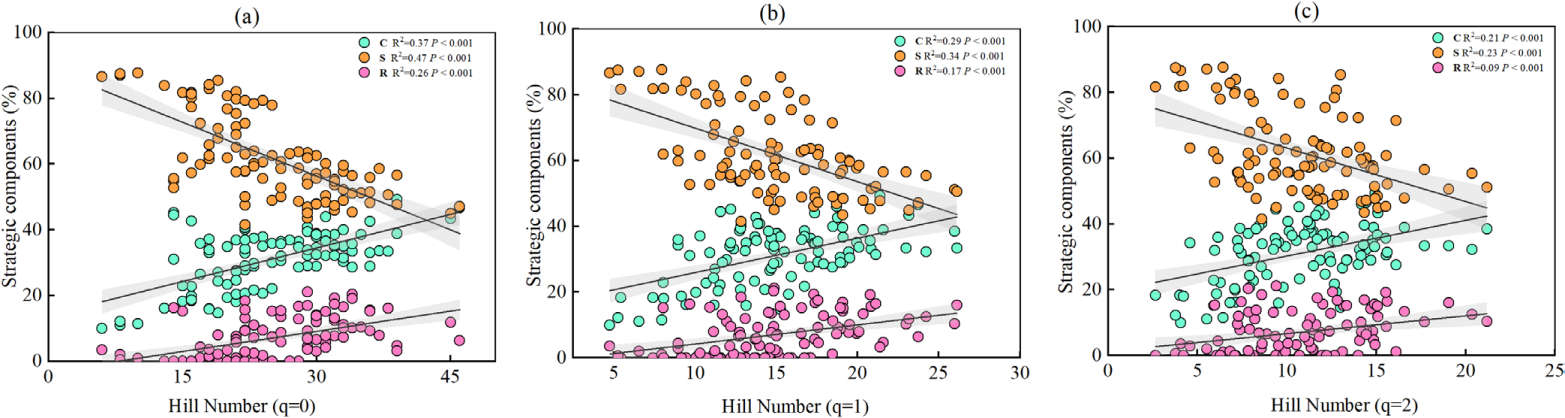
The varieties of CSR components of woody plants with a diversity of Hill numbers (a, *q* = 0; b, *q* = 1; c, *q* = 2). The solid lines indicate a significant correlation at *p* < 0.05, while the dashed lines indicate a nonsignificant correlation at *p* > 0.05.

The community‐level CSR components in the surveyed plots showed significant correlations with woody species diversity (Figure 3a–c). Specifically, the component S was negatively correlated with diversity, whereas the components C and R were positively correlated (*p* < 0.001). These relationships were consistent across Hill numbers (*q* = 0, 1, 2); as species diversity increased, components C and R increased significantly, while component S decreased. All results revealed a clear trade‐off between the stress tolerance strategy and the strategies for competition and ruderal, which were significantly associated with increased species diversity of woody plants during karst vegetation succession.

### 4.3 CSR Strategies Associated With Leaf Stoichiometric Traits of CNP

**FIGURE 4:**
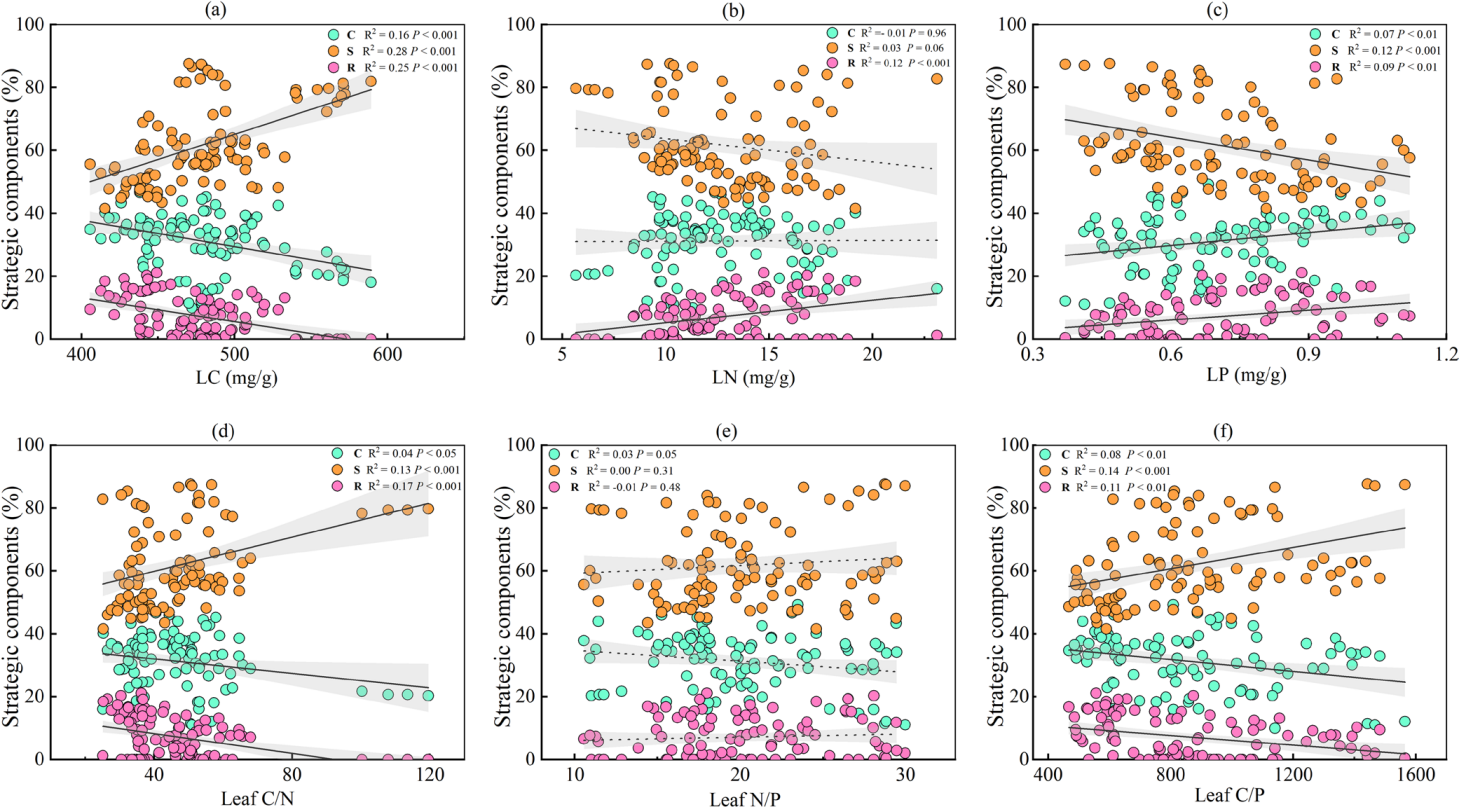
The varieties of CSR components with macronutrients of CNP (a‐c) and stoichiometric ratio. The solid lines indicate a significant correlation at the *p* < 0.05 level, while the dashed black lines indicate a nonsignificant correlation at the *p* > 0.05 level.

The community‐level CSR components and leaf CNP stoichiometric traits were significantly correlated across the surveyed plots (Figure 4a–f). Component S was significantly positively correlated with LC, leaf C/N, and leaf C/P (Figure 4a,d,f), while it was significantly negatively correlated with LP (Figure 4c). The components C and R were significantly positively correlated with LP (Figure 4c), while they were significantly negatively correlated with LC, leaf C/N, and leaf C/P (Figure 4a,d,f). Meanwhile, the component R showed a significant positive correlation with LN (Figure 4b). There was no significant relationship between strategy components of CSR and leaf N/P (Figure 4e). These results revealed a trade‐off between the stress tolerance strategy and the strategies for competition and ruderal, which was associated with leaf CNP stoichiometric traits during karst vegetation succession.

### 4.4 CSR Strategies Associated With Species Diversity, Leaf Functional Traits and Soil Properties

**FIGURE 5:**
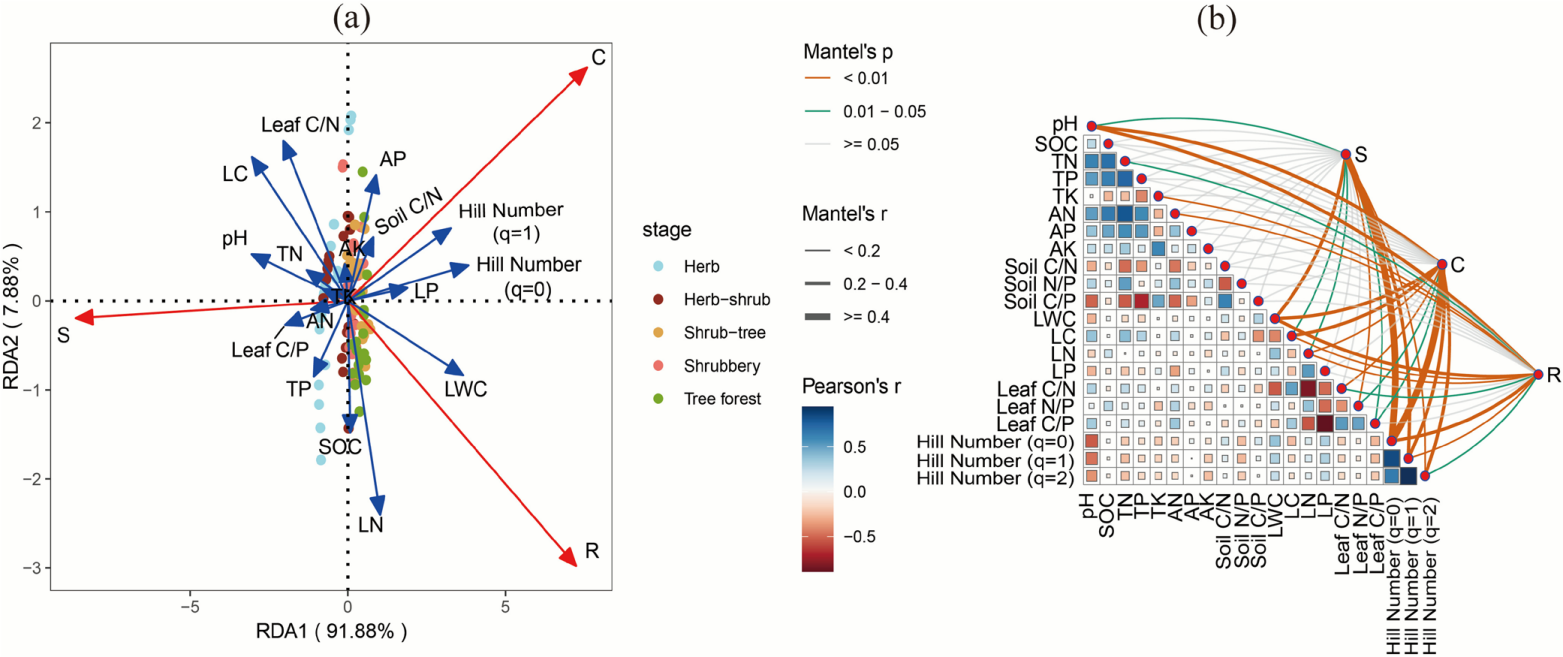
Redundancy analysis (a) and Mantel's test (b) for CSR components associated with species diversity of woody plants and functional traits with soil properties.

Redundancy analysis (RDA) revealed that the species diversity of woody plants, leaf functional traits, and soil properties collectively explained a significant portion of the variation in CSR components. The overall RDA model was statistically significant (permutation test, *p* < 0.001) and explained 67.00% of the variance (adjusted R‐squared), with the first and second axes capturing 91.88% and 7.88% of the constrained variation (Figure 5a). Hill number (*q* = 0) (48.05%), LWC (44.79%), and Hill number (*q* = 1) (35.71%) were identified as the primary explanatory variables. The CSR components exhibited distinct correlation patterns with environmental factors. Component C was positively associated with Hill numbers (*q* = 0, *q* = 1), soil C/N ratio, AP, AK, LP, and LWC, while negatively correlated with soil pH, TP, TN, TK, AN, and leaf C/P ratio. Component S was positively linked to pH, TP, TN, TK, AN, LC, and leaf C/N ratio but inversely related to AP, LP, soil C/N ratio, Hill numbers (*q* = 0, *q* = 1), and LWC. Component R showed positive correlations with Hill number (*q* = 0), SOC, LWC, LN, LP, and TP but negative associations with pH, TN, LC, and leaf C/N ratio. The influence of these factors shifted with succession. In early succession (herb and herb‐shrub stages), CSR strategies were primarily associated with LC, leaf C/N ratio, leaf C/P ratio, soil pH, and TP. In later stages (shrub‐tree, shrubbery, and tree forest), the strategies were mainly explained by Hill numbers (*q* = 0, *q* = 1), LWC, LP, LN, AP, and the soil C/N ratio. Mantel's analysis further confirmed significant correlations between CSR components and Hill numbers (*q* = 0, *q* = 1, *q* = 2), pH, LWC, and LC (Figure 5b). Collectively, these findings indicated that the species diversity of woody plants, particularly Hill numbers (*q* = 0, *q* = 1), along with LWC and LC, were the key factors associated with CSR strategies during karst vegetation succession.

### 4.5 The Relative Importance of Soil Properties, Leaf Functional Traits and Species Diversity in CSR Strategies

**FIGURE 6:**
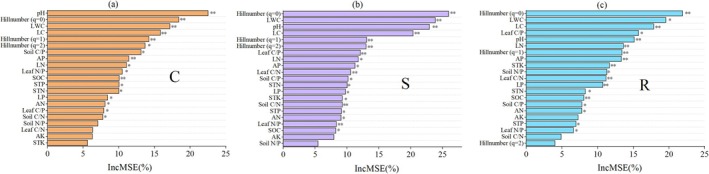
Relative cumulative importance of woody species diversity, leaf functional traits, and soil properties associated with CSR components.

The random forest model was employed to assess the relative importance of species diversity of woody plants, leaf functional traits, and soil properties to CSR strategies (Figure 6). Specifically, it identified Hill number (*q* = 0), soil pH, LWC, and LC as the most influential factors. For the C strategy, the three factors with the highest relative importance were soil pH, Hill number (*q* = 0), and LWC, with IncMSE of 22.50%, 18.41%, and 17.17%, respectively. Regarding the S strategy, the top three were Hill number (*q* = 0), LWC, and soil pH, with IncMSE of 25.97%, 23.86%, and 22.94%, respectively. As for the R strategy, the top three were Hill number (*q* = 0), LWC, and LC, with IncMSE of 21.92%, 19.56%, and 17.86%, respectively. In addition, the other factors also exhibited differing levels of relative importance for the CSR strategies. Overall, these results highlighted the dominant importance of Hill number (*q* = 0), soil pH, LWC, and LC on CSR strategies during karst vegetation succession. Specifically, soil pH was the most important factor for the C strategy, while Hill number (*q* = 0) was the most important factor for the S and R strategies.

## 5 Discussion

### 5.1 Shift in CSR Strategies During Vegetation Succession in the Karst Ecosystems

This study revealed clear clustering of CSR components on the C–S side across successional stages, with a pronounced bias toward the S vertex at both individual and community levels. This pattern indicates the predominance of the S strategy, followed by C and R strategies during the progress of karst vegetation succession. Moreover, a gradual shift in strategy components was observed, characterized by a decrease in component S and an increase in components C and R from the herb stage to the tree forest stage (Figure 2g). This indicates a trade‐off among CSR strategies through a shift from stress tolerance dominance toward competition and reproduction during karst vegetation succession. In our findings, the woody plant community exhibited a gradual transition from S strategies toward C and R strategies over the course of succession. This finding contrasts with the conventional view proposed by (J. P. Grime 1987, 1988), which posits that secondary succession should be initiated by R strategies, followed by C strategies dominating the intermediate stages, and ultimately succeeded by S strategies in the late phase. The discrepancy may stem from our focus on woody plants, which are not the dominant life form in the early herb and herb‐shrub stages. Our results align with several recent studies. For instance, Wen et al. (2022) suggested that woody plants usually employ S selection or CS followed by an inconspicuous R‐selection, exhibiting high‐stress tolerance to harsh environments by a finding on increasing C strategy coupled with a decreasing S strategy during tropical lowland rainforest succession. Similarly, Chen et al. (2023) observed an increase in C strategy species accompanied by a decrease in S strategy species as forests age, indicating the strategic trade‐off and strategy shift of C and S. These findings support our hypothesis (H1) that the early stages of karst vegetation succession are dominated by the S strategy, with a gradual shift toward C and R strategies as succession advances.

Wang et al. (2004) suggested that plants in karst regions inhabit harsh habitats with shallow, barren, calcium‐rich soils and acute water deficits, with vegetation degradation and the emergence of secondary vegetation being primarily caused by limited resource availability. These persistent stress pressures obliged plants to increase tolerance by the S‐selection for survival in strategy and to compete for limited nutrition by the C‐selection (Wen et al. 2022; Yang et al. 2024). Cerabolini et al. (2010) further indicated that S‐selection may occupy a broader range on the trait axis than C‐selection, reflecting more extensive adaptability under karst habitat limitations. This could explain the dominant S‐strategy observed across all successional stages in our study. Furthermore, component S had the highest proportion, while R had the lowest (Figure 2g). This indicates that S‐ and R‐selection represent two extremes in a strategic trade‐off of resource investment, prioritizing conservation for survival and allocation to reproduction. This finding is aligned with the leaf economics spectrum proposed by Wright et al. (2004). Recently, Hu et al. (2023) revealed the S‐strategy primarily governed plant individuals and communities with a competitive C‐strategy. Similarly, Yang et al. (2024) demonstrated that the C‐ and S‐strategy dominated the ecological strategy spectrum in karst‐natural forest regeneration from the mid‐successional stage to the climax community stage. These findings support our results showing that the predominant S strategy followed by the C and R strategies governed the vegetation succession in degraded karst ecosystem.

We employed the space‐for‐time substitution to explore the shifts in CSR strategies of woody plants during karst vegetation succession. Although this method has certain limitations, its underlying assumptions often conflict with site‐specific variations in disturbance history, microclimate, and soil properties (Buma et al. 2017; Lovell et al. 2023). Nevertheless, this approach has largely confirmed Grime's predictions (J. Grime 2001; Grime and Pierce 2012). When appropriately applied, the space‐for‐time method enhances the understanding of short‐term vegetation dynamics (Foster and Tilman 2000) and reveals critical successional information (Hobbs et al. 2007). It provides an essential framework for this study, allowing us to propose theoretical insights into strategy shifts during vegetation restoration in degraded karst ecosystems.

### 5.2 The Trade‐Off of CSR Strategies Associated With Plant Diversity and Functional Traits

Zanzottera et al. (2020) found that plant functional traits variation and CSR strategies at the community level shaped habitat structure along succession gradients in an alpine environment. Their study showed that, compared to single plant traits, CSR strategies allowed a more precise functional interpretation of alpine vegetation along the succession gradient and enabled the identification of realized functional niches within alpine communities, suggesting that the CSR framework is a powerful tool. This trait‐based approach can provide an operational assessment of niche for a potentially large number of species, making it possible to understand and predict species niche shifts under environmental changes (Violle and Jiang 2009). This indicates that trade‐offs in CSR strategies are associated with plant functional traits and species diversity. In our study, the ecological strategies of CSR showed a significant trade‐off between the S and the C and R. This trade‐off was associated with changes in Hill numbers (*q* = 0, 1, 2; Figure 3a–c) and with variations in leaf macronutrient contents of C, N, P, and their stoichiometric ratios of C/N, N/P, C/P (Figure 4a–f). These findings align with Zanzottera et al. (2020) in suggesting that trait trade‐offs between communities can experience regional‐scale adaptations due to local environmental conditions and that CSR strategies enable a more precise functional interpretation of alpine succession. Furthermore, they validate our hypothesis (H2) that there is a trade‐off between ecological strategies in karst plant communities, which is accompanied by changes in species diversity and plant traits. The increased C‐selection but decreased S‐selection with diversity increase, possibly due to species diversity playing an essential biotic driver in ecosystem function restoration (Tilman et al. 2014). Han, Xu, et al. (2022) suggested that low‐stress environments in later successional stages typically intensify competitive interactions among species through biotic filtering, implying preferentially supported survival and growth of competitively superior species.

Yang et al. (2024) suggested that improved soil conditions for plant survival and growth in the late successional stage contributed to more fertile and competitive conditions, leading to a decrease in the S strategy and an increase in the C strategy. This finding was consistent with the observed increase in species richness with increasing nitrogen availability during succession (Table S4), indicating that microhabitat quality improvement facilitates the competitive coexistence of more species. Therefore, high species diversity in later successional stages predicts the development of more complex community structures. These structures create more favorable microhabitats, which may lead to a higher proportion of component C, ultimately performing the joint role in plant trait variations such as the macronutrients of CNP and species diversity, as also noted by Chen et al. (2023). In addition, MacArthur and MacArthur (1961) demonstrated that vegetation in the later succession often exhibited increasing structural heterogeneity compared to early stages. Forests with longer stand ages tended to generate more litter, thereby enhancing the soil organic horizon (Castillo‐Figueroa and Posada 2025b). Litter significantly influenced ecosystem dynamics, such as soil food webs and nutrient fluxes during succession (Brown and Lugo 1990; Krishna and Mohan 2017). As succession progressed, increased litter input and more stable microclimatic conditions led to greater richness and abundance of soil fauna (Castillo‐Avila et al. 2025). These organisms play vital roles in ecosystem functioning by regulating litter decomposition and organic matter mineralization (Lavelle 1996; Heděnec et al. 2022), which may provide niche opportunities for R‐strategy species. Therefore, the woody plant communities in our study gradually showed a more substantial increase in R‐selection (Figure 2f). This can be attributed to the expansion of functional niche space, as observed by Zanzottera et al. (2020).

Furthermore, increased plant diversity altered the CSR pattern, manifested as opposing trends of component S relative to components C and R (Figures 3 and 4). This shift indicates a strategic trade‐off, probably due to functional trait differentiation in phenotype and macronutrients of CNP, which is demonstrated by varying values across different stages (Tables S2 and S5). Leaf macronutrients (C, N, P) and their ratios (C/N, C/P) reflect a plant's capacity for photosynthetic product storage and resource acquisition and utilization (Elser 2006). High C/N and C/P ratios indicate greater production with resource conservation, reflecting acquisitive and fast‐growing traits associated with the leaf economics spectrum (Figure 5a), as documented by Wang et al. (2023). Correspondingly, our study demonstrated that strategic trade‐offs of CSR associated with species diversity and leaf functional traits simultaneously contribute to plants' economic returns. This provides integrated evidence that combines CSR theory and the leaf economics spectrum.

### 5.3 Strategy Mechanisms of Degraded Vegetation Succession of Karst Ecosystem

The karst ecosystem generally presents a deficit of nitrogen and phosphorus (Shen et al. 2022), further impacting plant trait development (Zhong et al. 2018). Consistent with this, our study found that plant species in early successional stages exhibited lower LA and SLA, and higher LDMC compared to later stages (Table S2). This set of traits profiles aligns with a strategy of high S‐selection and low R‐selection (Figure 2f), supporting the theoretical framework of J. P. Grime (1977). As succession progressed, the functional trait values of woody plants showed directional changes alongside increasing species diversity. Meanwhile, the early successional stage of Herb and Herb‐shrub had lower diversity in richness and dominance (Table S4), along with low LA and high trait variation but high LDMC (Table S2). Tilman et al. (2014) suggested that plant variation in functional traits indicates resource partition and niche differentiation, which suggests that diversity alters trait adaptation strategically in niche space differentiation. In the early Herb stage, the low abundance and dominance of woody plants, along with their low LA and SLA, indicated reduced photosynthetic performance and light capture capacity. This led to their lower competitiveness and reproductive output, which was manifested as R‐ and C‐selection compared to later stages. At the same time, these plants had to cope with habitat stress, probably pressured by the availability of soil nitrogen and phosphorus.

Koerselman and Meuleman (1996) suggested that the plant N/P ratio reflects nutrient limitation: a ratio below 14 indicates nitrogen (N) limitation, above 16 indicates phosphorus (P) limitation, and a range between 14 and 16 signals simultaneous N and P limitation. The mean leaf N/P ratio across all stages in our study was 20.26, indicating the substantial P limitation in the karst ecosystem. Notably, the Herb stage presented the highest N/P ratio of 22.30 (Table S5). Fujita et al. (2014) found that plants from P‐limited habitats would invest less in sexual reproduction. Therefore, plants under low diversity adopted low reproduction by low R‐selection, possibly attributed to P limitation, as indicated by our results. Meanwhile, random forest also showed that leaf N/P ratio contributed more to components C and S than to component R (Figure 6a–c). The decrease in N/P ratio as succession progressed (Table S5) suggested a promotion of reproductive capacity and led to an increase in component C while decreasing component S. Consequently, nutrient P probably was the crucial factor in maintaining competition and reproduction during karst vegetation succession.

In addition, the soil pH and leaf water content (LWC) were also the key contributors to CSR strategies (Figure 6a–c). This was likely because of the variation from alkaline to acidic soil (Table S6) by increasing acidic exudates from roots or microbes in reducing pH, as well as the long‐term effects of drought on karst habitats, which stemmed from strong water leakage through extensive rock fissures (Liu et al. 2022). Therefore, it is worth exploring the mechanisms underlying the effects of biological exudates and water stress in vegetation successional dynamics strategically in future research. However, TP, AP, and C/N, N/P, and C/P of soil were not significantly related to CSR components (Figure 6a–c) or showed lower IncMSE% relative to leaf functional traits and species diversity of woody plants. We attribute this to the high heterogeneity of nutrient distribution in karst habitats, which weakens the relative contribution of nutrient availability to CSR strategies. Nevertheless, our study provides theoretical insights into the successional dynamics underlying the restoration of degraded vegetation. It elucidates how shifts in CSR strategies, associated with changes in species diversity and trait composition, support sustainable vegetation recovery in degraded ecosystems.

## 6 Conclusions

This study demonstrated a distinct strategic trade‐off, with a shift from stress‐tolerant (S) dominance to increasing contributions from competitive (C) and ruderal (R) strategies as succession progressed in degraded karst ecosystems. This transition was closely associated with increased woody species diversity and changes in key leaf functional traits and partial soil properties. The dominance of the S strategy in early successional stages underscores the harsh environmental constraints of karst habitats, while the gradual shift toward C and R strategies. There were strategic trade‐offs of CSR associated with species diversity and leaf functional traits, embodied in S relative to C and R strategies. Our study provides valuable theoretical insights into the successional mechanisms underlying vegetation restoration in degraded ecosystems and emphasizes the dynamic ecological strategies that enhance species diversity and change functional trait compositions, supporting sustainable vegetation recovery in degraded ecosystems.

## Author Contributions

**Menglin Lei:** formal analysis (equal), visualization (equal), writing – original draft (equal). **Kaiping Shen:** formal analysis (equal), writing – review and editing (equal). **Tingting Xia:** writing – review and editing (equal). **Xu Han:** writing – review and editing (equal). **Hongchun Chen:** data curation (equal). **Yan Zhao:** data curation (equal). **Xihong Yang:** formal analysis (equal), investigation (equal). **Jiahao Xiao:** formal analysis (equal), investigation (equal). **Fulin Wang:** formal analysis (equal), investigation (equal). **Ying Zhao:** formal analysis (equal), investigation (equal). **Weijie Li:** supervision (equal). **Yuejun He:** conceptualization (equal), funding acquisition (equal), methodology (equal), supervision (equal), writing – review and editing (equal).

## Conflicts of Interest

The authors declare no conflicts of interest.

## Supporting information


**Data S1:** ece372808‐sup‐0001‐Supinfo.docx.

## Data Availability

The dataset used in this study is available at the following: https://doi.org/10.6084/m9.figshare.30373492.
