# Supplementary material for: Shifts and Trade‐Offs of Ecological Strategy Associated With Species Diversity and Functional Traits During Vegetation Succession Progress in Karst Ecosystem
Source: Ecol Evol. 2025 Dec 23;15(12):e72808. doi: 10.1002/ece3.72808 (PMC12724010; doi:10.1002/ece3.72808)
Supplement: Supplementary file 1 — Data S1: ece372808‐sup‐0001‐Supinfo.docx. [file ECE3-15-e72808-s001.docx]

**Supplementary Material**

**Table S1 The different succession stages with their judgment basis and the community characteristics in respective stages.**

| **Stages** | **Criteria for stages** | **Vegetation situation and community properties** |
| --- | --- | --- |
| **Herb** | Community structure and layer; species composition; the relative abundance and relative coverage of herbs and woody plants; plant property in photosensitivity. | This was an herb community that experienced an accidental fire six years ago originated from a shrub grass community, according to visiting local villagers, and then it was closed under management by the forestry administration. This was the early stage in the karst second succession process. The community was opened with only one layer characterized by an average height of 0.4m, dominated by herbs with woody plants in relative abundance < 5% and coverage < 1% for each plot. There were 119 species, including 74 herbs with 45 woody plants from all plots statistically. At this stage, most of them were pioneer cheliophytes such as herbs of *Miscanthus sinensis* Andersson and *Pogonatherum crinitum* (Thunb.) Kunth and *Arthraxon hispidus* (Thunb.) Makino, woody of *Pyracantha fortuneana (Maxim.)* Li. and *Berberis thunbergii* DC. and *Myrsine africana* Linn. |
| **Herb-shrub** | Community structure and layer; species composition; the relative abundance and relative coverage of herbs and woody plants, and plant property in photosensitivity. Remaining plants from the previous stage and new plants are replaced. | Vegetation restoration took about 16 years, after experiencing unexpected fires from a local survey of the forestry administration. Subsequently, grazing and logging were prohibited due to the implementation of the rocky desertification project by the government in the past two decades through closure measures. There was a transitive feature composed of herb and shrub plants, approximately shared relative coverage from 40% to 60%, respectively, which was listed as the Herb-shrub stage. There was one layer for the plant community by average height of 1m approximately via woody saplings or small woody plants, while it was still not closed. Most plants are sun-positive and light-loving. A total of 133 species, including 73 herbs and 60 woody plants, were found in this stage, such as herbs of *Miscanthus sinensis Andersson and Pogonatherum crinitum (Thunb.) Kunth, Imperata cylindrica* (L.) Beauv, *Dicranopteris pedata* (Houttuyn) Nakaike. Woody of *Campylotropis macrocarpa* (Bge.)*, Pyracantha fortuneana (Maxim.)* Li, *Coriaria nepalensis* Wall. |
| **Shrubbery** | The dominance of woody plants, community height, and two community structural layers, shade-tolerant plants. Species numbers and diversity increased distinctly. | The restoration of shrub vegetation in this stage took at least 40 years and has been under enclosure and management since the 1980s, originating from the natural restoration of barren mountain ruins under the elimination of interference, according to a forestry administration interview. In this stage, two layers of community structure could be divided into the shrub and herb layers. Shrubs dominated the community with an average height of 1.9m, dividing two layers of shrub and herb plants in a plot. If the shrub plants dominate the community, simultaneously differentiated into two layers of community structure, when the average height of the community approaches 1.9m, which reflects a significant height difference compared to the Herb and Herb-shrub stages and Shrub-tree and Tree forest stage, the community was judged the Shrubbery stage. Meanwhile, the relative density of tree species was approximately < 10%, and it was also listed Shrubbery stage. Shade-tolerant plants increased, and the community closed. This stage had a total of 181 species, woody plant 95 and herbs 86. Main plant species included woody of *Itea yunnanensis* Franch, *Pyracantha fortuneana (Maxim.)* Li, *Stachyurus obovatus* (Rehder) Cheng*.* and *Viburnum chinshanense* Graebn*.*, herbs of *Miscanthus sinensis* Andersson, *Carex cruciata* Wahlenb, *Imperata cylindrica* (L.) Beauv. |
| **Shrub-tree** | The tree-transitional plants are growing in a community. The community height increased relative to the shrubbery stage, but was lower behind the stage through the visualized observation. | The vegetation at least took 60 years by continuous protection, but the specific origin remained unclear according to the manager and villagers. It is a transitional stage with a small number of tree plants in community appearance with a closed stand. Three layers may be divided into an indistinct tree layer and obvious shrub and herb layers in vertical structure. The relative density of trees ranges approximately from 10% to 60%, it was categorized into the Shrub-tree transition. 158 plant species were involving woody plants of 90 and herbs of 68. The main tree species were *Quercus acutissima* Carr*, Carpinus pubescens*Burkill*, Platycarya strobilacea* Sieb. et Zucc, with an average height of 8.5m. Meanwhile, this stage still retained partly plant species from the shrubbery stage, especially the shrub species *Itea yunnanensis* Franch still dominated the shrub layer through field observation. |
| **Tree forest** | The community becomes more complex in its vertical structure. Larger trees upper canopy layer dominate the whole community by coverage and height. Vines and shade-tolerant plants are present under the canopy layer. Generally, three layers or more complex. | The forest vegetation was typically distributed in karst mountains in this region, making it difficult to determine the origin time by our visit. Simultaneously, community structure could be divided into three layers of trees and shrubs and herbs. The species numbers and individuals of larger trees were distinctly more than the shrub-tree stage, dominating forest community by height. The average heights of tree plants were 14.5m in all plots. The relative density of trees ranges exceeds 60%, this community is classified as the Tree-forest stage. Some species of woody and herb plants were the same to other stages, however some vines and shade-tolerant plants such as lichen and moss increased compared to shrub-tree stage, which made community more complex. The species numbers were 177 involving 71 herbs and 106 woody statiscally from all plot survey. |

**Table S2 Leaf functional traits in community-weighted means of different succession stages**

| stage | LA (cm²) | SLA (cm²/g) | LDMC (%) |
| --- | --- | --- | --- |
| Herb | 13.43±15.58c  (116.01) | 40.34±3.98d  (9.87) | 42.87±2.26a  (5.27) |
| Herb-shrub | 16.16±8.74c  (54.08) | 43.54±7.17cd  (16.47) | 42.02±2.48a  (5.9) |
| Shrubbery | 27.37±9.75b  (35.62) | 46.74±7.58c  (16.22) | 38.21±3.14b  (8.22) |
| Shrub-tree | 35.79±12.62a  (35.26) | 51.44±6.28b  (12.21) | 37.67±4.57b  (12.13) |
| Tree forest | 38.2±15.52a  (40.63) | 62.03±8.51a  (13.72) | 38.72±2.19b  (5.66) |
| Total | 26.19±16.05  (61.28) | 48.82±10.15  (20.79) | 39.9±3.68  (9.22) |

Note: Different lowercase letters represent significant differences in leaf traits between different stages (*P* < 0.05); The data in the figure is the mean ± standard error. The number in brackets is the coefficient of variation.

**Table S3 CSR components for plant individuals in different vegetational stages**

| stage | Component C (%) | Component S (%) | Component R (%) |
| --- | --- | --- | --- |
| Herb | 19.26±15.02d  (77.97) | 74.22±16.32a  (21.98) | 6.51±10.58d  (162.42) |
| Herb-shrub | 22.92±15.08c  (65.81) | 69.35±17.28a  (24.92) | 7.73±11.15d  (144.25) |
| Shrubbery | 27.47±14.18b  (51.61) | 59.64±17.59b  (29.49) | 12.89±12.63c  (97.99) |
| Shrub-tree | 29.90±14.40a  (48.16) | 53.00±17.15c  (32.36) | 17.10±12.47b  (72.92) |
| Tree forest | 29.88±13.83a  (46.28) | 51.09±15.97d  (31.27) | 19.02±12.48a  (65.63) |
| Total | 26.84±14.86  (55.38) | 59.41±18.83  (31.70) | 13.75±12.97  (94.35) |

Note: Different lowercase letters represent significant differences in components between different stages (*P* < 0.05); The data in the figure is the mean ± standard error. The number in brackets is the coefficient of variation.

**Table S4 Hill numbers (q=0, 1, 2) of different succession stages.**

| stage | q=0 | q=1 | q=2 |
| --- | --- | --- | --- |
| Herb | 15±4.55e | 11.246±4.20c | 8.28±3.98b |
| Herb-shrub | 21±2.83d | 12.809±2.50bc | 9.29±2.38b |
| Shrubbery | 30±4.56b | 17.367±3.48a | 12.62±3.31a |
| Shrub-tree | 25±5.70c | 14.529±3.33b | 10.38±2.80b |
| Tree forest | 34±5.16a | 19.016±4.15a | 13.10±4.03a |

Note: Different lowercase letters represent significant differences in Hill number between different stages (*P* < 0.05); The data in the figure is the mean ± standard error.

**Table S5 Leaf chemical properties in community-weighted means of different succession stages**

| stage | LC (mg/g) | LN (mg/g) | LP (mg/g) | leaf C:N | leaf N:P | leaf C:P |
| --- | --- | --- | --- | --- | --- | --- |
| Herb | 497.88±36.51a  (7.33) | 13.3±4.21a  (31.65) | 0.64±0.15bc  (5.27) | 45.16±10.41bc  (23.05) | 22.30±4.71a  (21.12) | 940.7±261.36ab  (27.78) |
| Herb-shrub | 500.79±52.74a  (10.53) | 10.49±3.43b  (16.47) | 0.60±0.12c  (32.7) | 64.10±26.08a  (40.69) | 18.04±3.59c  (19.9) | 984.76±252.52a  (25.64) |
| Shrubbery | 489.89±26.99a  (5.51) | 11.39±1.34b  (16.22) | 0.75±0.24ab  (11.76) | 49.81±7.92b  (15.9) | 18.56±4.52bc  (24.35) | 890.17±294.77ab  (33.11) |
| Shrub-tree | 457.02±28.46b  (6.23) | 13.73±2.62a  (12.21) | 0.76±0.21a  (19.08) | 61.02±3.53a  (23.13) | 20.95±5.91abc  (28.21) | 789.34±346.44bc  (43.89) |
| Tree forest | 452.53±29.25b  (6.46) | 14.79±1.67a  (13.72) | 0.77±0.11a  (11.29) | 36.35±5.06d  (13.92) | 21.47±4.48ab  (20.87) | 714.67±141.54c  (19.80) |
| Total | 479.62±40.98  (8.54) | 12.74±3.22  (20.79) | 0.70±0.18  (25.27) | 46.98±16.73  (35.61) | 20.26±4.9  (24.19) | 863.93±280.73  (32.19) |

Note: Different lowercase letters represent significant differences in leaf traits between different stages (*P* < 0.05); The data in the figure is the mean ± standard error. The number in brackets is the coefficient of variation.

**Table S6 Soil properties of different succession stages**

| stage | Herb | Herb-shrub | Shrubbery | Shrub-tree | Tree forest |
| --- | --- | --- | --- | --- | --- |
| pH | 7.22±0.41a  (5.74) | 7.17±0.45a  (6.25) | 6.82±0.43b  (6.27) | 7.07±0.66ab (9.33) | 5.59±0.51c (9.08) |
| SOC (mg/g) | 45.91±11.42b (24.88) | 50.44±7.66ab (15.19) | 51.2±11.18ab (21.83) | 56.03±20.1a (35.87) | 49.22±10.04ab (20.39) |
| TN (g/kg) | 1.97±0.74c (37.79) | 2.16±0.97bc (44.92) | 1.82±1.07c (58.81) | 2.78±1.38ab (49.70) | 1.06±0.54d (51.14) |
| TP (g/kg) | 0.52±0.18bc (35.32) | 0.79±0.37a (46.94) | 0.61±0.29abc (46.72) | 0.66±0.34ab (51.92) | 0.44±0.22c (48.85) |
| TK (g/kg) | 10.78±2.53a (23.47) | 6.55±2.71b (41.34) | 5.63±3.99b (70.89) | 6.62±5.33b (80.55) | 6.42±7.6b (118.35) |
| AN (mg/kg) | 287±65.71ab (22.90) | 308.41±73.89ab (23.96) | 263.73±84.55bc (32.06) | 321.82±128.43a (39.91) | 226.45±79.46c (35.09) |
| AP (mg/kg) | 2.59±1.61b (62.02) | 5.52±2.93a  (53.1) | 6.6±4.93a  (74.78) | 7.51±5.65a (75.29) | 2.14±2.21b (103.01) |
| AK (mg/kg) | 102.81±22.21ab (21.60) | 115.03±32.94ab (28.64) | 90.98±40.3b (44.3) | 124.52±44.32a (35.60) | 93.66±61.51b (65.68) |
| Soil C:N | 24.83±4.65c  (18.74) | 28.32±12.9bc (45.54) | 48.35±56.63b  (117.12) | 22.55±5.38c (23.84) | 71.69±58.63a  (81.78) |
| Soil N:P | 3.79±0.52a (13.83) | 3.05±1.08b (35.51) | 2.8±1.38b  (49.16) | 4.31±0.73a (16.86) | 2.52±1.01b (40.01) |
| Soil C:P | 94.6±22.75b  (24.05) | 79.25±39.27b (49.55) | 99.62±48.41b (48.60) | 96.83±28.31b (29.24) | 145.84±92.02a (63.10) |

Note: Different lowercase letters represent significant differences in leaf traits between different stages (*P* < 0.05); The data in the figure is the mean ± standard error. The number in brackets is the coefficient of variation.
